# Duration of Perioperative Antibiotic Prophylaxis in Open Fractures: A Systematic Review and Critical Appraisal

**DOI:** 10.3390/antibiotics11030293

**Published:** 2022-02-23

**Authors:** Niels Vanvelk, Baixing Chen, Esther M. M. Van Lieshout, Charalampos Zalavras, T. Fintan Moriarty, William T. Obremskey, Michael H. J. Verhofstad, Willem-Jan Metsemakers

**Affiliations:** 1 Trauma Research Unit, Department of Surgery, Erasmus MC, University Medical Center Rotterdam, 3000 CA Rotterdam, The Netherlands; n.vanvelk@erasmusmc.nl (N.V.); e.vanlieshout@erasmusmc.nl (E.M.M.V.L.); m.verhofstad@erasmusmc.nl (M.H.J.V.); 2 Department of Trauma Surgery, University Hospitals Leuven, 3000 Leuven, Belgium; baixing.chen@student.kuleuven.be; 3 Department of Development and Regeneration, KU Leuven—University of Leuven, 3000 Leuven, Belgium; 4 Department of Orthopaedic Surgery, Keck School of Medicine, University of Southern California, Los Angeles, CA 90033, USA; zalavras@usc.edu; 5 AO Research Institute Davos, 7270 Davos, Switzerland; fintan.moriarty@aofoundation.org; 6 Department of Orthopaedic Surgery and Rehabilitation, Vanderbilt University Medical Center, Nashville, TN 37232, USA; william.obremskey@vumc.org

**Keywords:** fracture-related infection, duration, antibiotic, prophylaxis, open fracture

## Abstract

Fracture-related infection (FRI) remains a serious complication in open fracture care. Adequate surgical treatment and perioperative antibiotic prophylaxis (PAP) are key factors influencing the outcome. However, data concerning the optimal duration of PAP is scarce. The aim of this systematic review was to provide an overview of current evidence on the association between PAP duration and FRI in open fractures. A comprehensive search on 13 January 2022, in Embase, Medline, Cochrane, Web of Science and Google Scholar revealed six articles. Most studies compared either 1 day versus 5 days of PAP or included a cut-off at 72 h. Although prolonged PAP was not beneficial in the majority of patients, the variety of antibiotic regimens, short follow-up periods and unclear description of outcome parameters were important limitations that were encountered in most studies. This systematic review demonstrates a lack of well-constructed studies investigating the effect of PAP duration on FRI. Based on the available studies, prolonged PAP does not appear to be beneficial in the prevention of FRI in open fractures. However, these results should be interpreted with caution since all included studies had limitations. Future randomized trials are necessary to answer this research question definitively.

## 1. Introduction

Open fractures remain a major challenge in orthopaedic trauma surgery [1]. They are often associated with considerable bone damage including periosteal stripping, soft-tissue trauma and severe contamination. This enables bacteria to breach the damaged skin barrier and adhere to non-living surfaces (i.e., implants) [2,3]. Therefore, compared to closed fractures, they have a higher risk of developing a fracture-related infection (FRI). Overall, infection rates after internal fixation range from 1 to 2% in the case of closed fractures, and up to 25–30% in the case of severe open injuries. This risk increases with greater injury severity as classified by Gustilo and Anderson (GA) [4].

It is well-known in healthcare that our focus should be on prevention rather than improving treatment strategies [5,6]. With respect to the management of open fractures, this can be achieved with adequate surgical treatment (i.e., debridement, fracture stabilization, early soft tissue coverage) and systemic perioperative antibiotic prophylaxis (PAP) [7]. Many of these current strategies, such as the type of soft tissue coverage, timing of surgery, and application of local antibiotics, are however still under debate [8,9].

A particularly controversial and important topic is the use of PAP. While there is consensus that PAP lowers infection rates, especially when started as soon as possible after the injury occurs, the duration of systemic PAP remains controversial [10]. Contemporary guidelines state that PAP should be provided for no longer than 24 h in GA type I and II injuries [10]. In GA type III open fractures, PAP is advised to be discontinued after 72 h or 24 h after wound closure, whichever comes first [10]. These guidelines, however, are based on a handful of clinical studies, which still creates uncertainty related to the optimal duration of PAP and therefore heterogeneity in prevention protocols worldwide. Recent international surveys among orthopaedic trauma surgeons indeed concluded that the majority of surgeons continue PAP longer than recommended [1,11], which leads to an important antibiotic overuse [12]. Although PAP overuse may be attributed to the fact that open fractures are often complex injuries with a high FRI risk, it seems that the primary reason for divergent treatments is the lack of well-conducted studies establishing the optimal PAP duration [9].

For the abovementioned reasons, the aim of this systematic review was to investigate the association between PAP duration and FRI in open fractures and provide an overview of currently available evidence on this topic.

## 2. Materials and Methods

All relevant aspects of the Cochrane Handbook for Interventional Systematic Reviews [13] were followed and the study was written according to the Preferred Reporting Items for Systematic Reviews and Meta-Analyses (PRISMA) statement [14].

### 2.1. Search Strategy

A comprehensive search was performed on 13 January 2022 in Embase, Medline, Cochrane, Web of Science and Google Scholar. With the help of a biomedical information specialist, a set of search strings was composed for each database (Supplementary Material File S1).

### 2.2. Inclusion/Exclusion Criteria

To be included, published studies were required to present original data with the primary aim to investigate the association between systemic PAP duration and FRI in skeletally mature patients that were operatively treated for an open fracture. Furthermore, studies should provide a minimum follow-up period of two months, for the majority of the patients (≥75%). Exclusion criteria were studies that focused on fractures of the skull and fractures of the cervical, thoracic and lumbar spine. In addition, published abstracts, conference posters, letters and articles in languages other than English were excluded.

### 2.3. Screening Process

**Figure 1:**
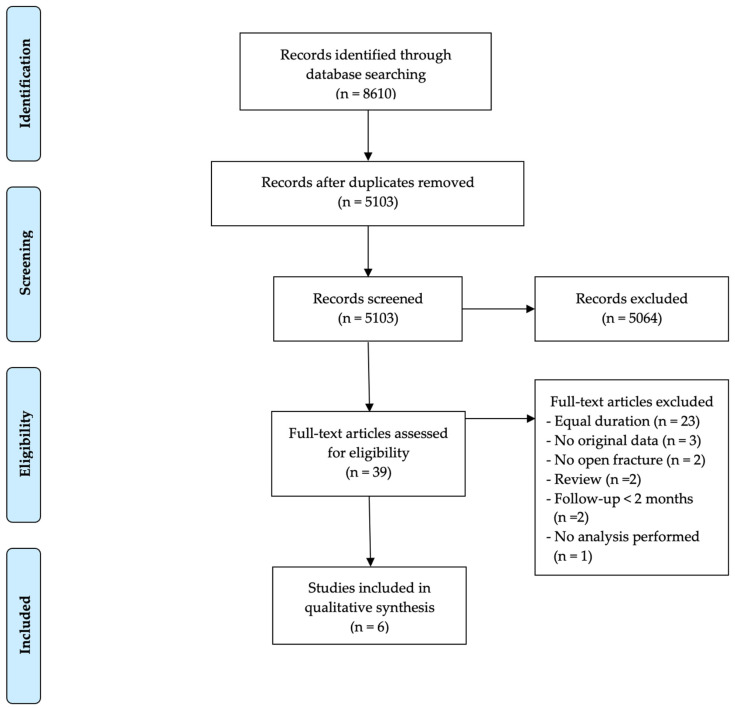
Outline of the search and selection process including exclusions and final number of studies included.

The search process is displayed in Figure 1. References were collected in EndNote and duplicates were removed. Screening was independently performed in a two-step process by two reviewers (N.V., W.-J.M.). A first selection was based on title and abstract screening. Afterwards, the full text of the remaining articles was evaluated for inclusion. In case the two reviewers did not reach a consensus, a third reviewer (C.Z.) was consulted. 

### 2.4. Data Extraction, Critical Appraisal and Quality Assessment

Data extraction and critical appraisal of the included trials was performed by two reviewers (N.V., B.C.). The data extraction sheet was developed a priori. Assessment for possible bias was performed using the ROBINS-I tool [15] for non-randomized studies and the RoB 2 tool [16] for randomized controlled trials. Articles for which consent could not be reached were discussed with a third, fourth and fifth reviewer (W.-J.M., C.Z., M.H.J.V.).

## 3. Results

Overall, 8610 references were collected in EndNote. After the exclusion of duplicates, 5103 articles remained. Based on title and abstract, 5064 articles were excluded. Of the remaining 39 articles, six (see Table 1) were included in this systematic review. The most important exclusion criterion at this step was the lack of a well-defined difference in PAP duration between patient populations (23 articles).

### 3.1. Study Design and Patient Characteristics

Two of the included articles presented the results of a randomized controlled trial (RCT). Dellinger et al. included 264 long bone and patella fractures, from 248 patients in a multicenter, double blinded RCT, which specifically aimed to investigate the effect of PAP duration on the infection rate in open fractures [17]. Carsenti-Etesse et al. divided 616 patients with tibia fractures from 43 centers into two groups based on antibiotic type and duration to determine the effectiveness of pefloxacin prophylaxis in infection prevention [19]. In another retrospective study, Dellinger et al. collected data from 240 patients (263 fractures) from three centers to investigate risk factors for the development of FRI [18]. In total, 84 of these patients (35%) had also been included in the previously mentioned RCT [17]. Patella fractures were left out of this retrospective study [18]. Declercq et al. (559 fractures) and Dunkel et al. (1492 fractures) included long bone and patella fractures in retrospective case-control studies to assess clinical variables associated with infection in open fractures with a focus on PAP duration [20,21]. Moreover, the latter also included fractures of the scapula and foot. Stennett et al. performed a secondary analysis of the Fluid Lavage of Open Wounds (FLOW) trial, including 2400 upper and lower limb fractures, to determine the association between PAP duration after definitive wound closure of an open fracture and FRI rates. Fractures of the hand and phalanges of the feet were excluded from this study [22].

### 3.2. Antibiotic Type and Duration

Most of the included studies compared either 1 day of PAP to 5 days or placed a cut-off at 72 h. In the RCT by Dellinger et al., patients were randomized into three groups to receive 1 day of cefonicid sodium, 5 days of cefonicid sodium or 5 days of cefamandole nafate (both being a second generation cephalosporin) [17]. In the non-randomized study by Dellinger et al., patients received mostly the same types of antibiotics. In this study, the duration was subdivided into less than 24 h, 1 day, 3 days and 4–5 days [18]. In the trial by Carsenti-Etesse et al., patients received different classes of antibiotics; the first group received a single dose of pefloxacin, a quinolone. The other group received 2 days of intravenous cefazolin, a first-generation cephalosporin, followed by 3 days of oral oxacillin, a penicillin [19]. In addition, prophylaxis against anaerobic infections by penicillin G or metronidazole/ornidazole was applied according to the protocol of each participating center. In the study by Stennett et al., all subjects received either a first- or second-generation cephalosporin. In patients with GA type III fractures, an aminoglycoside was added. In case of gross contamination, triple therapy including the two abovementioned antibiotics with additional penicillin was used [22]. Stennett et al. compared a PAP duration longer than 72 h to a duration of 72 h or less [22]. However, contrary to all other included studies, measurements of PAP duration did not start after the first dose was given, but after wound closure. Dunkel et al. and Declercq et al. described different PAP regimens [20,21]. In the majority of patients with a GA type I and II injury, a cephalosporin was administered as monotherapy (72% and 60% for Dunkel et al. and Declercq et al., respectively). The most common alternative was amoxicillin-clavulanic acid (4% and 8%, respectively). In GA type III fractures, there was a tendency to use an antibiotic with a broader spectrum, or a combination of a cephalosporin with aminoglycosides. Dunkel et al. compared 1 day, 2–3 days, 4–5 days and more than 5 days of PAP [20]. Declercq et al. placed a cut-off at 72 h [21].

### 3.3. Study Outcome

None of the four studies comparing PAP duration of up to 24 h with longer durations found a significant difference in FRI rates. In the RCT by Dellinger et al., no significant difference in FRI rates was found between 1 day of cefonicid sodium (13%), 5 days of cefonicid sodium (12%) and 5 days of cefamandole nafate (13%) in all open fracture types (*p* > 0.50) [17]. Likewise a separate analysis for GA type III fractures failed to show a protective effect of longer PAP durations [17]. In the non-randomized study by the same authors, no significant difference in infection rates was found when comparing a PAP duration of 8 h (15%) to 4–5 days (19%) by independent analysis of all fracture types (*p* > 0.50) and after multivariate correction for city/centers, fracture grade, fixation and fracture location (*p* = 0.90) [18]. In the study by Carsenti-Etesse et al., 2 days of cefazolin, followed by 3 days of oxacillin (8.0%) failed to show a protective effect when compared to 1 dose of pefloxacin (6.6%) in type I and II open fractures (*p* = 0.51) [19]. Dunkel et al. found no significant difference in FRI rates when comparing 1 day of PAP with 2–3 days (OR 0.6, *p* = 0.65), 4–5 days (OR 1.2, *p* = 0.21) or more than 5 days (OR 1.4, *p* = 0.26) in all fracture types [20]. A secondary analysis after isolating GA type III injuries also failed to show a significant difference [20]. 

In the studies placing a cut-off at 72 h, results varied. Declercq et al. found no difference in infection rates comparing a PAP duration of up to 72 h to longer than 72 h in all open fracture types (OR 3.61, *p* = 0.06), also after omitting GA type IIIB and IIIC fractures (OR 4.26, *p* = 0.07) [21]. Moreover, after adjustment for predictors associated with FRI, the authors found an association between PAP and FRI, showing that with every additional day PAP was continued, the odds of FRI increased (OR 1.11, *p* = 0.003) [21]. Stennett et al. found no significant difference in the initial unadjusted analysis. However, after stratification by wound contamination (according to the Orthopaedic Trauma Association (OTA) open fracture classification) and adjustment for numerous confounding variables including the GA type, they found different results for open fractures with either mild or severe contamination [22]. Increased odds of FRI were found when extending PAP duration beyond 72 h in fractures with mild contamination (OR 1.39, *p* = 0.12) [22]. A protective effect of extending PAP past 72 h was found in fractures with severe contamination (OR 0.20, *p* = 0.003) [22]. In open fractures with moderate contamination, no significant difference was found. 

### 3.4. Surgical Therapy

Even though most studies did not fully describe details, their treatment regimen was based on the principles of surgical debridement, irrigation and fracture stabilization. While both studies by Dellinger et al. mentioned that wounds were mostly left open for later closure [17,18], primary closure was pursued in most of the other studies. In the trial by Carsenti-Etesse et al., only open fractures that could be treated definitively (i.e., primary fixation and wound closure) were included [19]. In the study by Dunkel et al. priorities in management consisted of vascular repair, skeletal stabilization and copious irrigation. Second-look surgery was mostly performed after 48 h and definitive fracture fixation (with or without bone, skin and muscular grafting) was done at a later stage [20]. Since the article by Stennett et al. was based on data from the FLOW trial, mainly the irrigation procedure was well defined. Repeated irrigation and debridement procedures were performed until the wound was clean and definitive closure was pursued within 14 days of the initial surgery. The type of fracture stabilization was based on the surgeon’s preference [22]. In the article by Declercq et al., treatment was based on the surgeon’s preference, but at least consisted of immediate surgical debridement, irrigation and fracture stabilization [21]. Only three of the studies mention the use of local antibiotics [20,21,22], which was not standard of care. 

### 3.5. Outcome Description

The included articles used different definitions for infection. Dellinger et al. and Stennett et al. made a distinction between superficial and deep infection [17,18,22]. While Dellinger et al. formulated an original set of criteria for the diagnosis of FRI [17,18], Stennett et al. used the CDC criteria [22]. The definition used by Carsenti-Etesse et al. included microbiological and clinical data (e.g., presence of a fistula) [19]. In the study by Dunkel et al., the diagnosis of infection required the presence of pus combined with a surgical and antibiotic treatment [20]. The article by Declercq et al. used the FRI consensus definition [21]. 

### 3.6. Follow-Up Period

Follow-up periods varied widely. The trial by Carsenti-Etesse et al. had a follow-up period of three months [19]. The majority of patients included in the RCT by Dellinger et al. were followed for six months or more [17]. In the observational study, the same authors described a follow-up period of more than 3 months in 93% of the patients [18]. The retrospective studies by Dunkel et al. (2–120 months, median 35 months), Stennett et al. (12 months) and Declercq et al. (24 months) described longer follow-up periods [20,21,22]. However, Dunkel et al. excluded infections occurring after two months, considering them hospital-acquired and not related to the initial management of the open fracture.

## 4. Discussion

This systematic review includes two RCTs and four retrospective studies investigating the effect of PAP duration on the infection rate in open fractures. There were no studies that combined patients with open and closed fractures or patients with long bone and vertebral/skull fractures.

Based on the included studies, a prolonged PAP duration does not appear to be beneficial in the prevention of FRI. None of the studies comparing a PAP duration of up to 24 h with longer durations found a significant difference in FRI rates. Similar results were found in the studies using a cut-off at 72 h, which reported no benefit of prolonged PAP duration. On the contrary, they found that a PAP duration beyond 72 h may be associated with an increased FRI risk [21,22], except in severely contaminated open fractures [22]. The role of PAP duration in the presence of severe contamination, however, has only been investigated in a single study [22]. The findings of our systematic review are consistent with the most recent guidelines, which support discontinuing PAP after 24 h in GA type I–II injuries, and after 72 h or 24 h after wound closure (whichever comes first) in GA type III open fractures [10]. There was a previous attempt at a systematic review and meta-analysis [23]. However, most of the included studies in this review by Messner et al. did not have the effect of PAP duration on FRI rates as the primary study objective. Furthermore, many of our important inclusion criteria (e.g., long-term follow-up) were not taken into account. 

In the following sections we will describe the most important limitations encountered in the current literature. Moreover, we will present possible solutions that could aid in the development of future randomized controlled trials. 

### 4.1. Study Design

Although RCTs are already being performed to investigate the optimal PAP duration in prosthetic joint replacement [24], such studies are scarce in the field of orthopaedic trauma. In the present review, two RCTs on PAP duration in orthopaedic trauma patients could be included, both performed more than two decades ago. One of the reasons why such high quality trials are scarce, is the heterogeneity in the orthopaedic trauma patient cohorts. 

### 4.2. Antibiotic Type and Duration

While participants were divided into groups based on antibiotic duration in both RCTs, different types of antibiotics were included, making it difficult to interpret the results. The retrospective studies were more published recently, and the choice of antibiotic type, therefore, largely corresponds to contemporary guidelines [10]. In daily clinical practice, first and second generation cephalosporins are most often used, which was also mentioned by the retrospective studies of Dunkel et al. and Declercq et al. [20,21]. However, these authors still report a variety of antibiotic regimens. Future RCTs should be based on a single regimen to avoid possible confounding. 

Determining the optimal PAP duration in future RCTs is challenging as it is influenced by many factors. It seems advisable to at least include the durations already reported in current guidelines. In less severe injuries (GA type I–II) with only mild contamination, for example, a PAP duration of 24 h could be compared to a single dose of antibiotics. 

### 4.3. Classification

Multiple systems are currently being used to classify open fractures. The GA classification is widely incorporated in clinical practice, due to its ability to stratify open fractures according to infection risk [4]. Much more variation in soft tissue damage, fracture severity, bacterial contamination and consequently, surgical management strategies exist in GA type III injuries than in GA type I and II open fractures. This heterogeneity in injury severity and treatment will influence the outcome. The GA classification was mentioned in all articles included in this systematic review and most of the articles accounted for this factor by performing a secondary analysis isolating GA type III fractures. 

Variability in the interpretation of the GA classification has been described [25]. In response, the OTA proposed a new classification system [26]. While this offers a more detailed description of injuries and comparable interobserver agreement with respect to the GA classification, as of today, this classification is still not implemented on a large scale in daily clinical practice [27]. The OTA classification was only used in the study by Stennett et al. [22]. They classified the open fractures by degree of wound contamination and found decreased odds of FRI when continuing PAP beyond 72 h in severely contaminated injuries [22]. 

The severity of the open fracture—assessed by the GA or the OTA classification—is strongly associated with FRI and, therefore, a major confounding factor [28]. For this reason, it is critical to incorporate one of these classification systems in future research on PAP duration. This way a uniform group of patients can be defined to minimize the confounding effect of injury severity on infection rates. As there still seems to be a lot of debate on the optimal management of highly contaminated injuries (e.g., GA type III) and none of the studies showed a significant difference in outcome when comparing short versus long-term PAP duration in GA type I-II injuries, we suggest these latter groups would be an ideal area for a future RCT. 

### 4.4. Surgical Therapy

Fracture stability is of the utmost importance in the prevention of FRI. Instability leads to ongoing soft-tissue trauma, interruption of neo-vascularity and osteolysis of bone, which creates a supportive environment for bacterial proliferation [29]. However, in most studies included in this review, the surgical therapy (e.g., fracture stability) was poorly described. While all articles state fracture fixation as part of the initial management, the choice of stabilization was mostly not described in detail. As the type and timing of fracture fixation can have an influence on outcome, future RCTs should develop protocols that clearly delineate type and timing of fracture fixation and definitive soft tissue coverage as both play important roles with respect to the prevention of infectious complications [29,30]. 

In a systematic review and meta-analysis, Morgenstern et al. found a risk reduction of 11.9% of FRI associated with the use of local antibiotics in the management of open fractures [2]. While the authors state these results should be interpreted with caution due to the limited quality of the available evidence, the results suggest a positive effect of the use of local antibiotics in the prevention of FRI. Although some of the studies included in this systematic review mentioned the use of local antimicrobials, it was not consistent [20,21,22]. As the use of local antimicrobials could have an important influence on the outcome, it seems advisable that they are not included or included uniformly in future RCTs that focus on duration of PAP.

### 4.5. Outcome Description

Correctly defining complications is critical for every study. Of the six studies included in the present systematic review, only two used a standardized definition of FRI. Stennett et al. used the CDC criteria for the diagnosis of infection [22] and Declercq et al. described outcome based on the recently developed FRI consensus definition [21]. In 2018, this consensus definition for FRI was composed in response to a systematic review showing that only 2% of the RCTs on fracture fixation used standardized criteria to describe FRI [31,32]. Recently, Onsea et al. validated the confirmatory criteria of the FRI consensus definition [33]. Furthermore, in a retrospective cohort study by Sliepen et al., the authors compared the CDC criteria to the FRI consensus criteria and found that when the diagnostic criteria of the FRI consensus definition were used, 98.9% of FRIs could be captured, while only 49.8% of infections were diagnosed when using the CDC criteria [34]. Finally, in this systematic review, half of the included articles made a distinction between superficial and deep infections [17,18,22]. It has however previously been stated that the differentiation between both types is arbitrary and can only be confirmed by deep tissue sampling [35]. This is one of the reasons why this subdivision was not included in the FRI consensus definition. Based on the best available evidence, the use of the diagnostic criteria of the FRI consensus definition appears to be the preferable option for future clinical research.

### 4.6. Follow-Up Period

In this review, follow-up periods were mostly short with only two studies exceeding twelve months for all included patients. Furthermore, two articles were excluded because the follow-up period was too short or poorly described. In a study by Ondari et al., the follow-up period was limited to 14 days [36]. Reisfeld et al. stated that the authors reviewed the medical records of included patients until 30 days after the admission, but did not mention how many patients actually had follow-up [37]. Interestingly, only a single study reported a follow-up of 24 months for the whole patient population [21]. This suggests that late onset infections were probably missed in most studies. The importance of adequate follow-up has been demonstrated by Zalavras et al. [38]. In a recent retrospective study on the timing of FRI onset in patients with open fractures, the authors found that a follow-up period of 90 days captured 64% of FRIs, while 89% of the FRIs were captured after one year of follow-up [38]. Since missing late-onset infections will make an intervention appear better than it actually is, an adequate follow-up period is especially important in clinical research. We thus propose a minimum follow-up period of one year for all future studies on this topic.

### 4.7. Future Directions

Although PAP is an accepted strategy to prevent FRI, prolonged administration may contribute to the current problem of antimicrobial resistance (AMR). AMR is a major issue in clinical practice today and considered one of the important global health challenges of the twenty-first century [12]. Therefore, physicians are becoming aware of the importance of limiting antibiotic use [9,12]. Improving and following PAP guidelines will be a priority in the future to limit the increasing prevalence of multidrug-resistant pathogens [39]. High-quality trials are needed to establish the optimal protocols for the prevention of infection in open fracture care. This review presents possible solutions that could aid in the development of such trials (Table 2).

## 5. Limitations

The main limitation of this systematic review is the inclusion of only a small number of studies that investigate the association between PAP duration and FRI. Furthermore, the RoB-2 tool showed some concerns for the risk of bias in the RCTs, while the ROBINS-I tool demonstrated a moderate risk of bias in the non-randomized trials. Moreover, due to the limited number of patients included in the two RCTs, these were likely underpowered and drawing conclusions from the results should be done with caution. For these reasons and due to the presence of significant heterogeneity with respect to the studied patient cohorts, a valid meta-analysis could not be performed.

## 6. Conclusions

This systematic review demonstrates the lack of well-constructed studies investigating the effect of PAP duration on FRI. Based on the included studies, prolonged PAP duration does not appear to be beneficial in the prevention of FRI. However, due to important limitations, these results should be interpreted with caution and a specific cut-off in time after which PAP can safely be discontinued remains uncertain. The most important limitation is the large heterogeneity in the studied patient cohorts, with differences in study designs, antibiotic types, surgical treatment, fracture types and localizations, follow-up periods and outcome descriptions.

## Figures and Tables

**Table 1:** Characteristics of eligible studies.

First Author, Year of Publication	Study Design	Evidence Level *	Number of Patients (Fractures)	GA Type	Antibiotic Type and Duration	Study Outcome	Follow-Up Period
Dellinger, 1988 [17]	RCT	Some concerns	248 (264)	I, II, III	Group 1: 1 day of cefonicid sodium Group 2: 5 days of cefonicid sodium Group 3: 5 days of cefamandole nafate	No significant difference in infection rates between group 1 (13%), group 2 (12%) and group 3 (13%) (*p* > 0.50) No significant difference in infection rates between group 1 (24%), group 2 (22%) and group 3 (21%) after isolating GA type III fractures (*p* > 0.90)	2 months ^1^
Dellinger, 1988 [18]	Retrospective case-control ^+^	Moderate	240 (263)	I, II, III	Cefonicid sodium, cefamandole nafate, cefazolin sodium 8 h vs. 1 day vs. 3 days vs. 4–5 days	No significant difference in infection rates when comparing PAP duration of 8 h (15%) vs. 4–5 days (19%) by independent analysis of all patients (*p* > 0.50) No significant difference in infection rates when comparing PAP duration of 8 h vs. 4–5 days after multivariate correction for center, fracture grade, fixation method and fracture location (*p* = 0.90)	3 months ^2^
Carsenti-Etesse, 1999 [19]	RCT	Some concerns	616 (616)	I, II	Group 1: Single dose of pefloxacin Group 2: 2 days of cefazolin, followed by 3 days of oxacillin	No significant difference in infection rates between group 1 (6.6%) and group 2 (8.0%) (*p* = 0.51)	3 months
Dunkel, 2013 [20]	Retrospective case-control	Moderate	1290 (1492)	I, II, III	Multiple antibiotic types 1 day vs. 2–3 days vs. 4–5 days vs. more than 5 days	No significant difference in infection rates when comparing 1 day of PAP vs. 2–3 days (OR 0.6, *p* = 0.65), 4–5 days (OR 1.2, *p* = 0.21) or more than 5 days (OR 1.4, *p* = 0.26) No significant difference in infection rates when comparing 1 day of PAP vs. 2–3 days (OR 0.3, *p* = 0.95), 4–5 days (OR 0.6, *p* = 0.24) or more than 5 days (OR 1.7, *p* = 0.43) after isolating GA type III fractures	2 months ^3^
Declercq, 2020 [21]	Retrospective case-control	Moderate	502 (559)	I, II, III	Multiple antibiotic types Cut-off at 72 h	No significant difference in infection rates when comparing PAP duration of up to 72 h vs. more than 72 h in all injuries (OR 3.61, *p* = 0.06) or after omitting GA type IIIB and IIIC fractures (OR 4.26, *p* = 0.07) Adjusted for LASSO selected predictors, PAP duration was independently associated with infection (OR 1.11 for every one day increase in PAP duration, *p* = 0.003)	24 months
Stennett, 2020 [22]	Retrospective cohort	Moderate	2400 (2400)	I, II, III	Cephalosporin for all injuries. In type III injuries an aminoglycoside was added and in grossly contaminated injuries a penicillin was added Cut-off at 72 h	No significant difference in infection rates when comparing PAP duration of up to 72 h vs. more than 72 h (OR 0.96, *p* = 0.81) In open fractures with mild contamination, extending PAP duration past 72 h was associated with increased odds of infection (OR 1.39, *p* = 0.12) In open fractures with severe contamination, extending PAP duration past 72 h was associated with decreased odds of infection (OR 0.20, *p* = 0.003)	12 months

GA: Gustilo-Anderson; LASSO: Least Absolute Shrinkage and Selection Operator; OR: Odds Ratio; PAP: Perioperative Antibiotic Prophylaxis; RCT: Randomized Controlled Trial; * As evaluated by the Revised Cochrane Risk-of-Bias tool for randomized trials (RoB 2) or the Risk Of Bias In Non-randomized Studies assessment tool (ROBINS-I); ^+^ Retrospective analysis of data collected in two prospective studies; ^1^ Overall, 78% of the included patients had a follow-up period of more than 2 months; ^2^ Overall, 93% of the included patients had a follow-up period of more than 3 months; ^3^ Follow-up ranged from 2 to 120 months, but infections occurring after two months were excluded, considering them hospital-acquired and not related to the initial open fracture management.

**Table 2:** Suggestions for the development of future studies.

Focus Areas	Recommendations
Study design	Randomized controlled trial
Antibiotic type and duration	Use of a single antibiotic regimen in accordance with contemporary guidelines
Surgical therapy	Clearly defined surgical treatment strategy (e.g., immediate definitive fracture fixation and soft tissue closure)
Classification	Incorporation of a classification system to define a uniform group of patients (e.g., Gustilo-Anderson type I and II injuries)
Outcome description	Use of a standardized outcome definition (FRI consensus definition)
Follow-up period	Adequate duration of follow-up (minimum one year)

## Data Availability

Data is contained within the article.

## Supplementary Materials

The following are available online at https://www.mdpi.com/article/10.3390/antibiotics11030293/s1, File S1: Search strategy.
