# Supplementary material for: Duration of Perioperative Antibiotic Prophylaxis in Open Fractures: A Systematic Review and Critical Appraisal
Source: Antibiotics (Basel). 2022 Feb 23;11(3):293. doi: 10.3390/antibiotics11030293 (PMC8944527; doi:10.3390/antibiotics11030293)
Supplement: Supplementary file 1 [file antibiotics-11-00293-s001.zip › antibiotics-1589556-supplementary.pdf]

Supplementary Material

File S1. Search strategy.

Database searched	via	Years of coverage	References	After de-duplication
Embase	Embase.com	1971 - Present	4329	2506
Medline ALL	Ovid	1946 - Present	2173	2169
Web of Science Core Collection *	Web of Knowledge	1975 - Present	1610	290
Cochrane Central Register of Controlled Trials	Wiley	1992 - Present	298	63
Other sources: Google Scholar			200	75
Total			8610	5103

*Science Citation Index Expanded (1975-present) ; Social Sciences Citation Index (1975-present) ; Arts & Humanities Citation Index (1975-present) ; Conference Proceedings Citation Index- Science (1990-present) ; Conference Proceedings Citation Index- Social Science & Humanities (1990-present) ; Emerging Sources Citation Index (2015-present)

Embase

('infectious complication'/exp OR 'bacterium contamination'/de OR 'infection prevention'/de OR 'antiinfective agent'/exp OR 'antibiotic prophylaxis'/de OR (antibiotic* OR antibacterial* OR antimicrobial* OR antiinfectiv* OR antiseptic* OR ((anti) NEXT/1 (biotic* OR bacterial* OR infect* OR microbial* OR septic*)) OR ((infect* OR bacterial* OR bacterium* OR purulent*) NEAR/3 (prevent* OR prophylax* OR surgic* OR surger* OR operat* OR postsurg* OR postoperat* OR perisurg* OR perioperat* OR complicat* OR contaminat*))):ab,ti,kw) AND ('open fracture'/de OR (((compound* OR open* OR contaminat*) NEAR/6 (fractur*))):ab,ti,kw) NOT ((animal/exp OR animal*:de OR nonhuman/de) NOT ('human'/exp))

Medline

(exp Anti-Infective Agents/ OR Antibiotic Prophylaxis/ OR (antibiotic* OR antibacterial* OR antimicrobial* OR antiinfectiv* OR antiseptic* OR ((anti) ADJ (biotic* OR bacterial* OR infect* OR microbial* OR septic*)) OR ((infect* OR bacterial* OR bacterium* OR purulent*) ADJ3 (prevent* OR prophylax* OR surgic* OR surger* OR operat* OR postsurg* OR postoperat* OR perisurg* OR perioperat* OR complicat* OR contaminat*))).ab,ti,kw.) AND (Fractures, Open/ OR (((compound* OR open* OR contaminat*) ADJ6 (fractur*))).ab,ti,kw.) NOT ((exp animal/) NOT (human/))

Cochrane

((antibiotic* OR antibacterial* OR antimicrobial* OR antiinfectiv* OR antiseptic* OR ((anti) NEXT/1 (biotic* OR bacterial* OR infect* OR microbial* OR septic*)) OR ((infect* OR bacterial* OR bacterium* OR purulent*) NEAR/3 (prevent* OR prophylax* OR surgic* OR surger* OR operat* OR postsurg* OR postoperat* OR perisurg* OR perioperat* OR complicat* OR contaminat*))):ab,ti,kw) AND ((((compound* OR open* OR contaminat*) NEAR/6 (fractur*))):ab,ti,kw)

Web of Science
TS=(((antibiotic* OR antibacterial* OR antimicrobial* OR antiinfectiv* OR antiseptic* OR ((anti) NEAR/1 (biotic* OR bacterial* OR infect* OR microbial* OR septic*)) OR ((infect* OR bacterial* OR bacterium* OR purulent*) NEAR/2 (prevent* OR prophylax* OR surgic* OR surger* OR operat* OR postsurg* OR postoperat* OR perisurg* OR perioperat* OR complicat* OR contaminat*)))) AND ((((compound* OR open* OR contaminat*) NEAR/5 (fractur*)))) NOT ((animal* OR rat OR rats OR mouse OR mice OR murine OR dog OR dogs OR canine OR cat OR cats OR feline OR rabbit OR cow OR cows OR bovine OR rodent* OR sheep OR ovine OR pig OR swine OR porcine OR veterinar* OR chick* OR zebrafish* OR baboon* OR nonhuman* OR primate* OR cattle* OR goose OR geese OR duck OR macaque* OR avian* OR bird* OR fish*) NOT (human* OR patient* OR women OR woman OR men OR man)))

Google Scholar – top 200 relevant records
"antibiotic|antibacterial|antimicrobial|antiinfective|antiseptic prophylaxis"
"open|compound|contaminated fracture|fractures"
